# Method for Determining Contact Temperature of Tool Rake Face During Orthogonal Turning of Ti-6Al-4V Alloy

**DOI:** 10.3390/ma18132980

**Published:** 2025-06-24

**Authors:** Łukasz Ślusarczyk, Agnieszka Twardowska

**Affiliations:** 1 Department of Production Engineering, Faculty of Mechanical Engineering, Cracow University of Technology, Al. Jana Pawła II 37, 31-864 Krakow, Poland; 2 Institute of Technology, University of the National Education Commission, ul. Podchorazych 2, 30-084 Krakow, Poland; agnieszka.twardowska@uken.krakow.pl

**Keywords:** tool temperature, Ti-6Al-4V alloy, MATLAB PDE

## Abstract

This paper proposes a method for determining the contact temperature in the secondary shear zone. The input data include the results of the experimental tests of the orthogonal turning of a Ti-6Al-4V titanium workpiece using uncoated WC-Co tools with a flat rake face. The cutting force components were recorded using a piezoelectric dynamometer, a thermovision camera was used to record the temperature in the cutting zone, and a high-speed camera was used to record the chip-forming process. The independent variables included machining parameters, feed rate, cutting speed, and rake angle. A dual-zone thermomechanical cutting process model that accounted for the sticking and sliding areas was adapted for the identification of the heat flux in the chip–rake face contact zone. Then, based on the Shaw approach, the partition coefficients were determined for the contact temperature on the chip–tool tip contact. In addition, the results of the experimental tests allowed the determination of the relationship among the process parameters, friction coefficients, and the length of the contact of the chip with the tool rake face. A graphical visualization of the temperature distribution on the tool rake face was performed using the MATLAB PDE 3.9 software package. Although the application of the dual-zone model has been well presented in the literature, the results provided in this paper may be helpful in analyzing and modeling thermal phenomena in the secondary shear zone.

## 1. Introduction

Despite the constant development of various manufacturing processes, machining still plays the leading role in shaping machine parts. The continuously growing requirements of the industry require constant progress in the machining processes. Heat generation is a part of each machining process. This can lead to changes in the tool and workpiece material properties, particularly in the surface layer [1,2].

The extensive experimental research on tool wear and machined surface integrity in the high-speed turning of 18CrNiMo7-6 has been described in [3]. The authors described the wear process of coated cermet and coated carbide blades for turning speeds of V = 100, 160, and 200 m/min. Over the entire range of tests, the coated cermet tools had a significantly higher wear resistance. For speeds of V = 100 and 160 m/min, the coated cermet experienced oxidative wear, while the coated carbide tools were worn by coating peeling. However, at V = 220 m/min, both tools exhibited coating peeling and notch wear. Moreover, surface roughness, surface microhardness, and residual compressive stress increased with the progression of tool wear.

A significantly high temperature can reduce the hardness of tool materials and consequently the resistance to wear; as a result, the tool tip quickly becomes blunt [1,4,5,6].

Another issue is surface machining the workpiece in order to improve its integrity. In [7], the authors described an ultrasonic nanocrystal surface modification (UNSM) method to improve the surface quality of 316L workpieces made using the selective laser melting (SLM) method. The microhardness, compressive residual stress, and yield strength of the UNSM-treated sample were significantly improved compared to the control samples.

The determination of the temperature in the cutting zone is a significant issue that has been widely described in the literature. There are contact and contactless methods. Paper [8,9] describe the solutions used for contact temperature measurement. The authors have described the following measurement methods: Resistance Methods (RMs), thermocouple (TC/DTC), Thermophysical Processes (TPs), and Spectral Radiation Thermometry (SRT/TCP). The appropriate method should be chosen by the temperature measurement range, resistance to interference, response time, and ease of implementation [10].

A practical application of contact temperature measurement methods during the machining of Ti-6Al-4V alloy is described in [11]. In the study, the cutting temperatures were measured both by inserting thermocouples at various locations of the tool–chip interface using the tool–work thermocouple technique. The cooling effectiveness was tested by using atomization-based cutting fluid (ACF) spray.

The other group of methods used to measure the temperature in the cutting zone are contactless measurements. A paper [12] presents the adaptation of a thermovision device for the analysis of the temperature distribution in the cutting zone. The authors described the use of a high-resolution thermovision microscope to determine the temperature distribution on the chip flank during the orthogonal turning of AISI 1045 steel.

An article [13] presents the use of a novel near-infrared two-color pyrometer during the dry and wet turning of Ti-6Al-4V. The proposed solution eliminates the effect of object emissivity on the measured temperature. It has a good dynamic response and can show the temperature changes during machining without delays.

In [14], a high-speed thermovision camera was adapted to measure the temperature distribution in the cutting zone during the turning of AISI 1045 and AISI 4140 steel. The device allowed the recording of thermovision sequences at 328 fps at a resolution of 320 × 256 pixels. Arrazola et al. [15] used a thermovision camera to record the tool–chip contact temperature during the orthogonal turning of AISI 4140 steel and Ti6Al4V titanium alloy. The obtained experimental results were used to calibrate the cutting process simulation models. Despite the relatively easy adaptation of thermovision devices for temperature determination in the cutting zone, such methods have several significant shortcomings, such as the impossibility of using IR measuring devices during zone cooling or difficulties with the unequivocal estimation of the emissivity of the measured surfaces [16,17].

The next group of methods for the determination of the temperature in the cutting zone is the numerical calculation methods. Numerical calculation methods are alternatives for temperature determination in the cutting zone. Contemporary computer programs allow creating simulation models of cutting processes. Papers [18,19] describes the application of the Abaqus/Explicit (2016) calculation package and AdvantEdge (6.0) FEM software. These papers raises the important issue of building the material model by means of the correct selection of the coefficients in the Johnson–Cook constitutive equation. A paper [20] describes adaptations of the machining simulation model, which were later verified by experimental measurements. The AdvantEdge 3D (6.0) numerical calculation package was used to build the hard-turning simulation model. The built model allowed the determination of the residual stresses after the turning of AISI 52100 steel. The main difficulties in adapting simulation models involve the justification of the built models. According to [21,22,23], it is important to correctly build material models of the workpiece and the tool. The next important issue is the FEM mesh adaptation.

In [24], the coolant effects on the cutting temperature, surface roughness, and tool wear were investigated by using the developed virtual machining system. The cutting forces during the turning operations of Ti6Al4V alloy were accurately calculated in order to be used for the calculation of the cutting temperature and tool wear. The modified Johnson–Cook methodology was utilized to obtain the cutting temperatures along the machining paths. Then, the coupled Eulerian–Lagrangian (CEL) approach was investigated to predict and evaluate the effects of coolants on the cutting temperature in turning operations of Ti6Al4V alloy.

Paper [25] describe the stages of building the cutting process simulation models. Another approach involves the use of analytical calculation methods to estimate the temperature in the cutting zone. For the determination of the heat flux value on the chip–tool contact, the dual-zone model according to Zorev’s theory can be used. Studies [26,27] have used sticking and sliding theory in the secondary shear zone. Subsequently built were hybrid models to estimate the temperature in the cutting tool. The authors have used the MATLAB PDE software package. A paper [28] presents analytical methods of temperature determination in the primary and secondary shear zone. They are based on the J-C equations and the cutting mechanics according to Oxley. The analytically obtained temperature values were verified by experimental measurements using a thermovision camera.

## 2. Methodology

**Figure 1:**
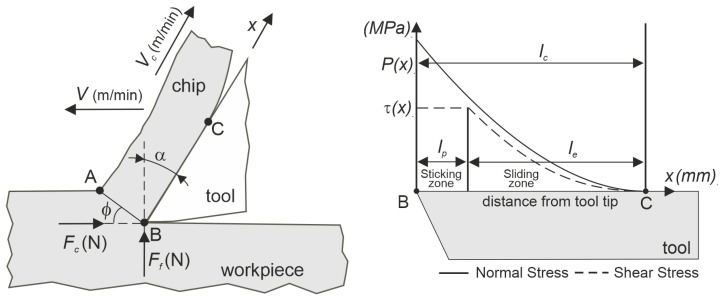
The distribution of the stresses in the secondary shear zone according to the dual-zone model [27].

The friction between the chip and the tool rake face is the dominating mechanism in the modeling of phenomena in the secondary shear zone. For orthogonal machining, Zorev proposed the dual-zone model to describe this phenomenon. The contact between the chip and the rake face is modeled in two areas: the first (at the tool tip) in the sticking zone, and the second is the area of sliding friction. The distribution of the stresses in the secondary shear zone according to the dual-zone model [27,29,30,31] is presented in Figure 1.

In the first area (sticking zone), the contact is plastic due to the high normal stresses exerted on the tool. However, the shear stress may not exceed the material shear flow stress $\tau_{1}$. In the second area (sliding contact), the normal stresses are lower, which are represented by sliding friction. The relationship between the normal stress and the shear stress is proportional and, according to Coulomb’s law of friction is, described by the sliding friction coefficient τ=μP, where μ is the friction coefficient, and P is the normal stress. The formulas below describe the relationships occurring in the dual-zone model for orthogonal turning. The distribution of the shear stresses in two areas on the rake face is defined as (1):

(1) $$\tau =\left\{\begin{array}{c} \;\;\;\;\;\;\;\;\tau_{1},\;\\ \mu P\left(x\right),\; \end{array}\right.\begin{array}{c} x\leq \;l_{p}\\ \;\;\;\;\;\;\;\;\;l_{p}\leq x\;\leq \;l_{c} \end{array}$$

where x (mm) is the distance from the tool tip along the rake face, l_p_ is the sticking length, and l_c_ is the total contact length. Based on [30,31],

(2) $$l_{p}=l_{c}\left(-{\left(\frac{\tau_{1}}{p_{0}\mu }\right)}^{\frac{1}{\zeta }}+1\right)$$

(3) $$l_{c}=h_{1}\frac{\zeta +2}{2}\frac{\sin\left(\phi +\lambda_{a}-\alpha \right)}{\sin\phi \cos\lambda_{a}}$$

where

(4) $$\lambda_{a}=\mathrm{atan}\left(\frac{F_{t}}{F_{c}}\right)+\alpha$$

As mentioned earlier and based on [27], it is assumed that the value of the shear stress in the sticking zone is identical to the material flow stress (plasticizing stress). Accordingly, the value of $\tau_{1}$ is determined using Formula (5).

(5) $$\tau_{1}=\frac{{(F}_{c}\cos\phi -F_{p}\sin\phi )\sin\phi }{\mathrm{bh}}$$

The normal stress in the sliding contact area is described by Formula (6).

(6) $$P(x)=P_{0}{\left(1-\frac{x}{l_{c}}\right)}^{\zeta }$$

where P_0_ is the normal stress at the tool tip, and ζ is the exponent of distribution. Based on [26,27], ζ = 2 is used for further calculations.

(7) $$P_{0}=\tau_{1}\frac{h(\zeta +1)}{l_{c}\sin\phi }\frac{\cos\lambda_{a}}{\cos(\phi +\lambda_{a}-\gamma_{0})}$$

The value of the shear angle ϕ is determined using the relationship

(8) $$\phi =\frac{\pi }{4}-\frac{\beta }{2}+\frac{\alpha }{2}$$

where α is the rake angle; β is the friction angle

(9) $$\beta =\mathrm{atan}\left(\frac{F_{f}}{F_{c}}\right)+\alpha$$

Two friction coefficients are determined: the apparent friction coefficient $\mu_{a}$, which is calculated from the total cutting forces acting on the tool rake face, and the coefficient of sliding friction μ, which is calculated from the cutting forces acting on the sliding area of the rake face.

The relationship between the coefficients is described by Formulas (10) and (11).

(10) μa=tan⁡(λa)=τ1P01+ζ1−τ1P0μ1ζ

(11) $$\frac{\tau_{1}}{P_{0}}=\frac{\zeta +2}{4\left(\zeta +1\right)}\frac{\sin\left(2\left(\phi +\lambda_{a}-\alpha \right)\right)}{({\cos\;\lambda_{a})}^{2}}$$

The division of the secondary shear zone into two areas determines the two values of the chip flow speed on the rake face. In the sticking zone, the chip flow speed is a function of distance x, and in the sliding contact area it is a fixed value. The relationship describing the chip flow speed on the rake face in the examined areas is presented below (12).

(12) $$V_{c}\left(x\right)=\left\{\begin{array}{c} V_{c0}{\left(\frac{x}{l_{st}}\right)}^{\omega_{c}}\;\;\;\;\;\;\;\;0\leq x\leq \;l_{p}\;\;\;\;\;\;\;\\ \;\;\;\;\;\;\;\;\;\;\;\;\;\;\;\;\;V_{c0}\;\;\;\;\;\;l_{p}<x\leq \;l_{c}\;\;\;\;\;\;\\ \;\;\;\;\;\;\;\;\;\;\;\;\;\;\;\;\;\;\;\;0\;\;\;\;\;\;\;\;\;x>l_{c}\;\;\;\;\;\;\;\;\;\;\;\;\;\; \end{array}\right.$$

The heat flux generated in the secondary shear zone is described using the relationship in (13).

(13) $$\dot{q}\left(x\right)=\left\{\begin{array}{c} \;\;\;\;\;\;\;\;\;\;\;\;\tau_{1}V_{c}\;\;\;\;\;\;\;\;0\leq x\leq \;l_{p}\;\;\;\;\;\;\;\\ \mu V_{c}P\left(x\right)\;\;\;\;l_{p}<x\leq \;l_{c}\;\;\;\;\;\;\\ \;\;\;\;\;\;\;\;\;\;\;\;\;\;\;\;0\;\;\;\;\;\;\;\;\;\;\;\;\;x>l_{c}\;\;\;\;\;\;\;\;\;\;\;\;\;\; \end{array}\right.$$

The heat partition ratio between the chip and the rake face is determined in accordance with the moving heat source model [31]. The knowledge of the total cutting energy flowing to the tool tip in the form of heat flux is necessary to determine the cutting temperature. In this theory, the moving heat source is assigned to the chip, so the heat partition ratio R_ch_ determines the percentage of heat flowing to the chip. On the other hand, the part (1 − R_ch_) is equal to the percentage of heat entering the tool tip. The heat partition ratio R_SH_ determined according to Shaw is presented in equation

(14) RSH=11+0.754λTλWAaNT

The thermal and geometrical properties of the contact surface are defined by the thermal conductivity of the machined material and the tool, where $\lambda_{W}$ and $\lambda_{T}$ are the thermal conductivities of the machined material and the tool, respectively. The thermal number $N_{T}$ is (Equation (15))

(15) $$N_{T}=\frac{V_{c}l_{c}}{2\alpha_{w}}$$

where $V_{c}$ is the chip speed (sliding), l_c_ is the contact length, $\alpha_{w}$ is the thermal diffusivity of machined material (chip). The average value of the shape coefficient $A_{a}$ is expressed by Equation (16), where m = $a_{p}$, and $\frac{m}{l}$ is the discriminant of the shape (elongation).

(16) $$A_{a}=\frac{2}{\pi }\left[{{sinh}^{-1}\left(\frac{m}{l}\right)+\left(\frac{m}{l}\right){sinh}^{-1}\left(\frac{l}{m}\right)+\frac{1}{3}{\left(\frac{m}{l}\right)}^{2}+\frac{1}{3}\left(\frac{l}{m}\right)-\frac{1}{3}\left\{+\left(\frac{m}{l}\right)\right\}\left\{1+{\left(\frac{m}{l}\right)}^{2}\right\}}^{0.5}\right]$$

**Figure 2:**
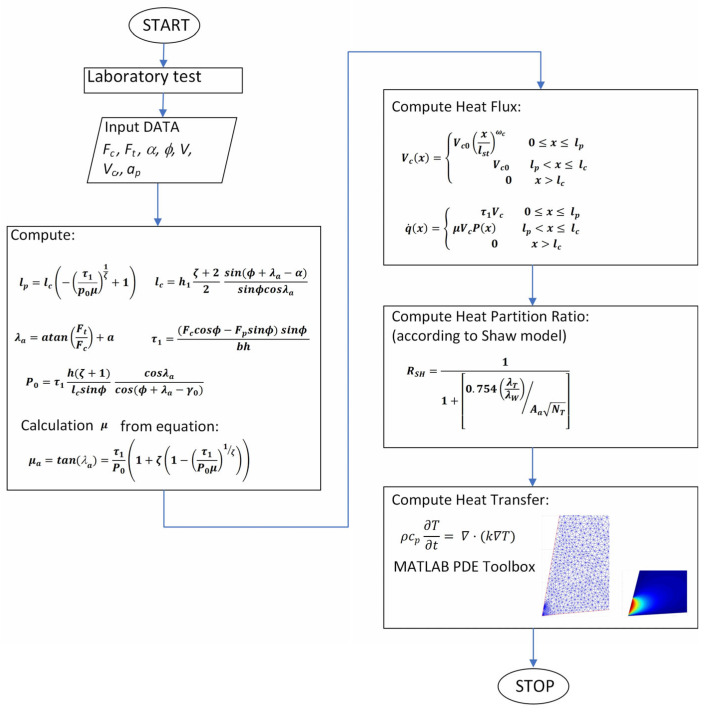
The diagram of the proposed method.

The diagram of the proposed method is presented in Figure 2. The input data were experimentally determined by the cutting strength components and the speed of the chip flow on the tool rake face. Then, in accordance with the diagram below, we determined the partitions of the heat flux entering the tool from the side of the rake face. There are methods described in the literature that allow the heat flux in the secondary zone to be determined and partitioned [31,32]. The proposed method utilizes experimentally determined cutting forces. The method is original and allows the quick estimation of the temperature on the tool rake face. Shaw’s model is used to determine the heat flux partitioning.

## 3. Materials and Methods

### 3.1. Materials

The experimental tests involved the orthogonal turning of a suitably prepared shaft made of Ti-6Al-4V alloy. The chemical composition of Ti-6Al-4V alloy is presented in Table 1.

### 3.2. Methods

**Figure 3:**
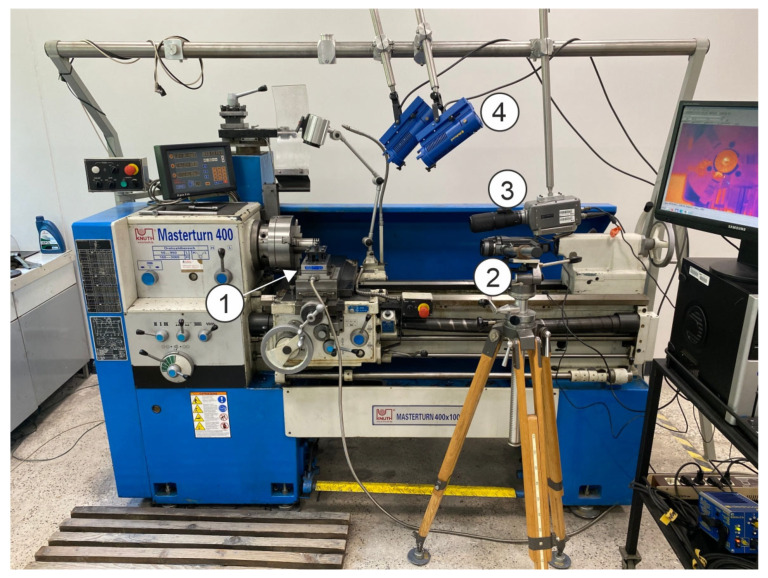
The test stand: 1—a piezoelectric dynamometer, 2—a thermovision camera, 3—a high-speed camera, 4—the lighting system.

The experimental tests of the orthogonal turning of Ti-6Al-4V alloy were conducted on a KNUTH Masterturn 400 precision lathe (KNUTH Machine Tools, Wasbek, Germany). The cutting force components were recorded during the tests, and a high-speed camera was used to record the chip-forming process, with a thermovision camera used to measure the temperature in the cutting zone. The test stand is shown in Figure 3.

The setup for measuring the cutting force components included a Kistler 9257B piezoelectric dynamometer installed on the lathe’s auxiliary slide and a Kistler 5070B charge amplifier (Winterthur, Switzerland). The obtained cutting force values were stored and analyzed on a PC with DynoWare software (version 2825A, Kistler Group, Winterthur, Switzerland). The measuring setup recorded the cutting forces at a frequency of 3000 Hz. The average values of the cutting forces were used in the further analyses. A PHANTOM v5.2 high-speed camera (Vision Research) with a fixed-focus lens (NIKON NIKKOR f = 200 mm) was used to record the chip-forming process, in particular to determine the speed of the chip flow on the tool rake face. The camera was running at 3000 frames per second, and the resolution was 512 × 512 px. A Dedolight Dedocool Colt 3 lighting system was used. The recordings were analyzed in specialist applications CineViewer and Tracker. A FLIR SC 620 thermovision camera (Teledyne FLIR, Wilsonville, OR, USA) with a fixed-focus lens with f = 38 mm and a computer with specialist software ThermaCam Researcher 2.1 were used to record and analyze the temperature distribution in the cutting zone. The camera was recording at a frequency of 30 Hz and a resolution of 640 × 480 px. The high-speed camera and the thermovision camera were installed on the lathe’s tailstock side and allowed the observation of the side of the chip flowing on the rake face.

**Figure 4:**
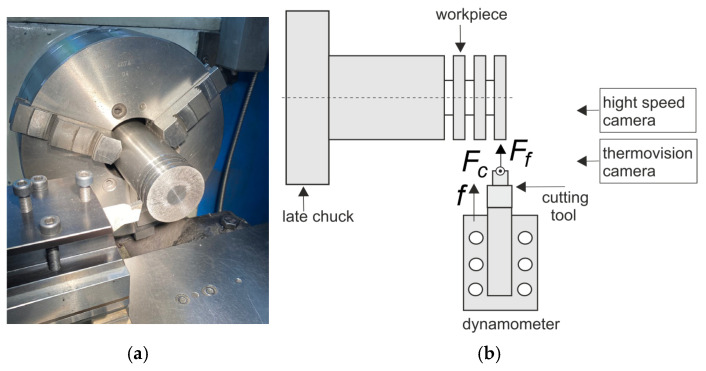
(**a**) The fixing of the dynamometer and the cutting tool; (**b**) a diagram of the recorded cutting force component.

The fixing of the dynamometer and the cutting tool is shown in Figure 4a. The orthogonal turning was performed at a constant cutting speed on a suitably prepared shaft. Two cutting force components, $F_{c}$ and $F_{f}$, which were the temperature in the cutting zone and the process of the chip formation and flow, were recorded during the tests, in accordance with the diagram in Figure 4b.

**Figure 5:**
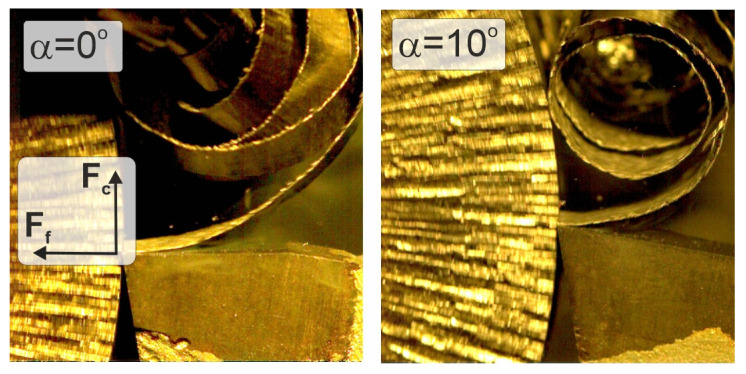
Chip-forming process recorded by high-speed camera.

The tests were performed with uncoated WC-Co with a flat rake face. The tool rake angles were α = 0° and α = 10°. The clearance angle and the inclination angle of the cutting edge were 5° and 0° in both cases. The geometry of the cutting tools used affected the chip form, which facilitated the experimental analysis of the chip flow on the rake face. The chip-forming process recorded by the high-speed camera for the two different tool geometries used in the tests is presented in Figure 5.

**Figure 6:**
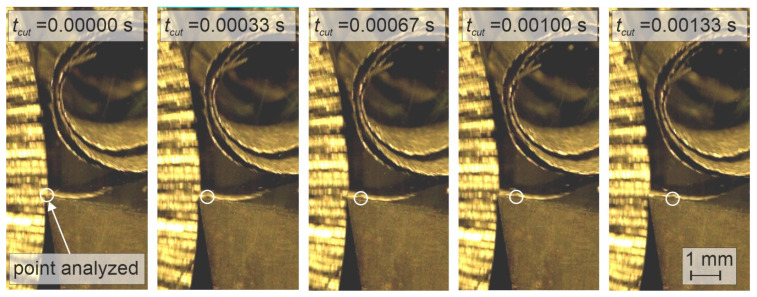
An exemplary sequence of the chip flowing on the rake surface of the tool.

In addition, the high-speed camera allowed tracing the characteristic point (marker) on the chip flowing on the tool face. The markers were made on the side of the workpiece by means of knurling. The time-lapse analysis of the cutting process using specialized software allowed the average chip flow speed to be determined (Figure 6).

**Figure 7:**
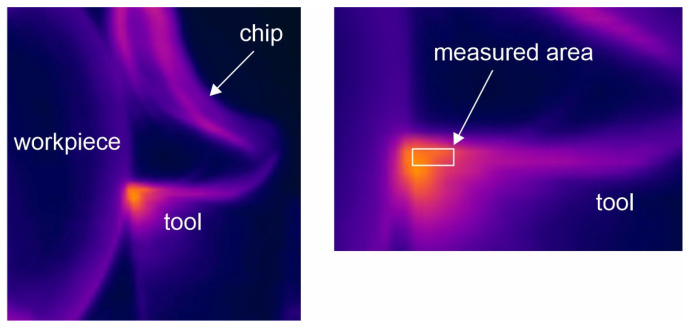
Example of the sequence recorded by the thermal imaging camera with the analyzed area marked.

Figure 7 presents an example of the thermovision image with a rectangle area. Based on the literature, the emissivity for the uncoated WC-Co tool was assumed to be ε = 0.48 [33]. Studies with a similar setup for contactless temperature measurement are described in [34].

**Figure 8:**
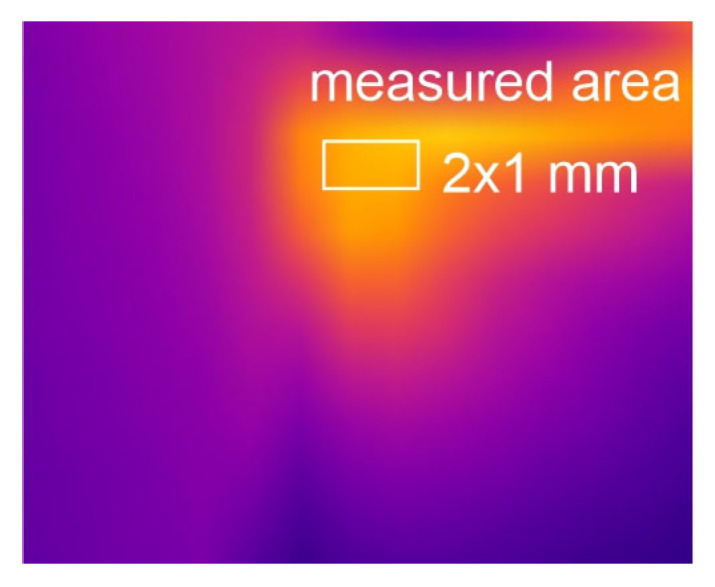
Recorded tool flank along with chip flowing.

In the experiments, the tool flank along and the chip flowing on it (Figure 8) were recorded with a thermovision camera. Such positioning of the thermovision camera guaranteed that the recording did not experience interference caused by the flowing chip. The thermovision recordings from each test were subjected to further processing. The stable course of the cutting process was used in the subsequent analyses.

The analysis of the obtained thermograms allowed the determination of the average contact temperature. The temperature was measured on the tool flank on a small area of 2 × 1 mm.

The range in the processing parameters during the tests is shown in the Table 2.

The feed rate and cutting sped were independent variables. The cutting depth was constant.

The cutting tests were performed according to the plan presented in Table 3.

## 4. Results

The average measured values of the cutting forces and the determined values of the shear angle and shear stress are presented in Table 4 below. The forces presented in the table are the average values from three tests, obtained during a stable cutting process.

**Figure 9:**
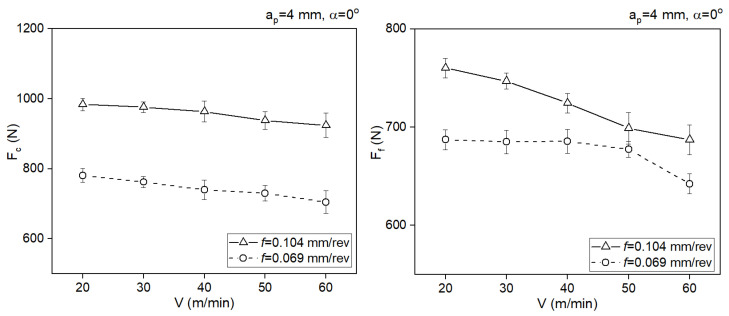
Impact of the cutting force on the values of the cutting force components *F_c_* and *F_f_*, *a_p_* = 4 mm, *α* = 0°.

**Figure 10:**
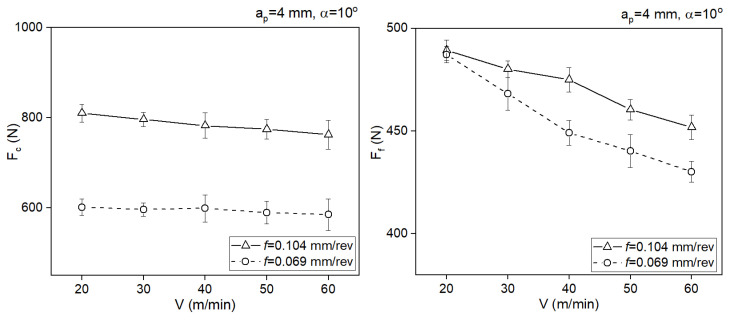
Impact of the cutting speed on the values of the cutting force components *F_c_* and *F_f_*, *a_p_* = 4 mm, *α* = 10°.

Figure 9 and Figure 10 present the cutting force components as a function of the cutting speed for two rake angle values. In both cases, the increase in the cutting speed slightly reduces the cutting forces components. A larger rake angle reduces the values of the forces.

**Figure 11:**
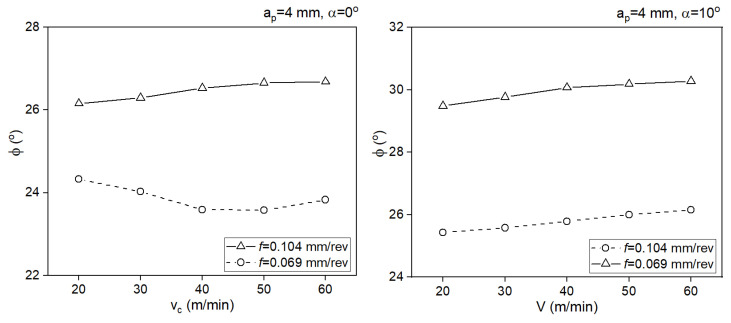
Impact of the cutting speed on the shear angle for two rake angle values.

Figure 11 presents the shear angle for two rake angle values.

Greater values of the shear angle were obtained for the larger cross-section of the cut layer. The positive tool rake angle also significantly increased the rake angle.

The experimentally determined average values of the chip flow speed and average contact temperature in the cutting zone are shown in Table 5 below. The maximum error in the thermovision measurements was ±8%.

**Figure 12:**
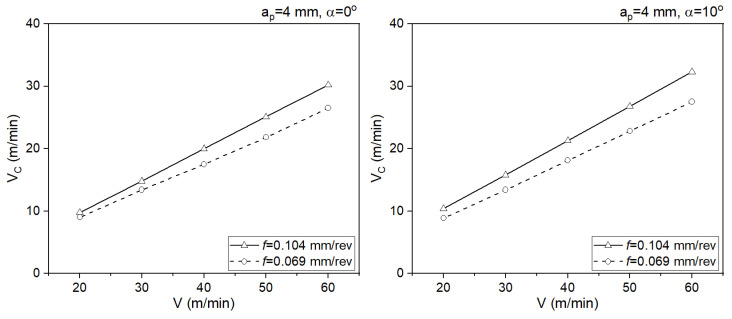
The impact of the cutting angle on the average values of the chip flow speed.

**Figure 13:**
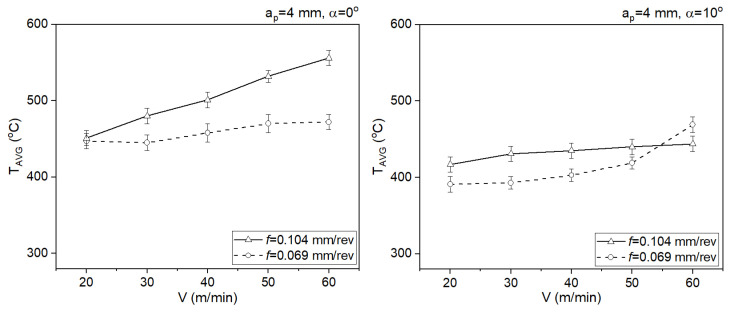
The impact of the cutting speed on the average values of the contact temperature.

In the case of tools with rake angles of α = 0° and α = 10°, the angle does not have a significant impact on the chip flow speed. In both cases, lower chip flow speeds are observed for the slower feed rates (Figure 12). The average values of the contact temperature were determined during the stabilized cutting process. Confidence intervals were added to each average value (Figure 13).

The values of the maximum normal stress and shear stress were determined using a calculation algorithm (Figure 2). The values of the sliding friction coefficient and apparent friction coefficient were determined based on formula (10) (Table 6). The algorithm was implemented in the MATLAB calculation environment.

**Figure 14:**
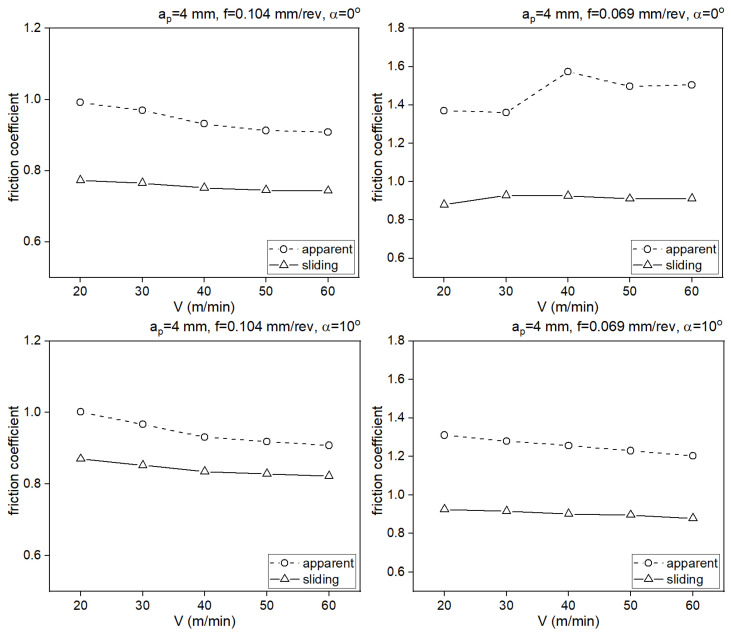
Impact of cutting speed on values of sliding friction coefficient and apparent friction coefficient.

The values of the sliding friction coefficient and apparent friction coefficient are presented in Figure 14. For each test, the values of the sliding friction coefficient were greater than those of the apparent friction coefficient. A similar relationship is described in the literature.

Table 7 presents the analytically determined length of the chip contact with the tool face, where $l_{c}$ is the total contact length, $l_{p}$ is the sticking length.

**Figure 15:**
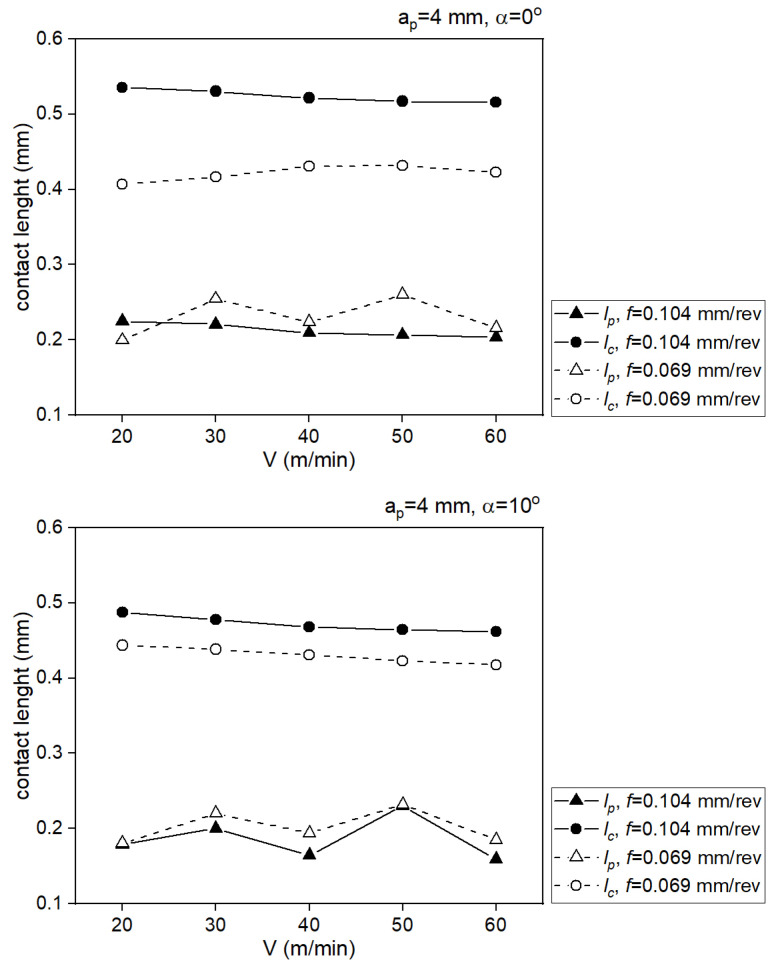
The impact of the cutting speed on the values of the length of the chip contact with the tool face.

The impact of the cutting speed on the values of the length of the chip contact with the tool face is presented in Figure 15.

Shaw’s heat partition ratio and the heat fluxes propagating to the cutting tool were determined using the workpiece and the tool’s thermo-mechanical properties according to Table 8.

The determined Shaw’s heat partition ratio and the heat fluxes entering the tool and the workpiece are given in the Table 9 below.

**Figure 16:**
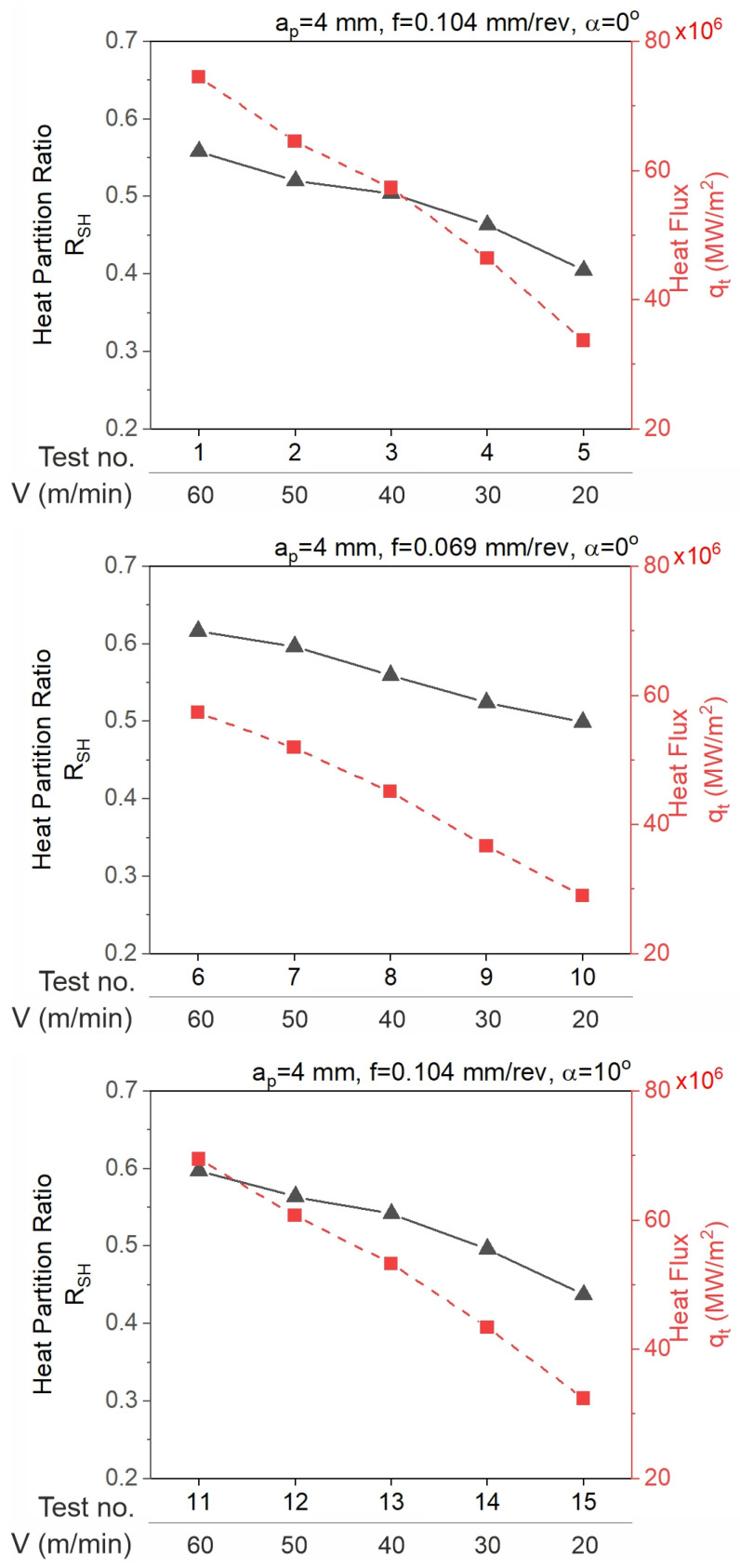

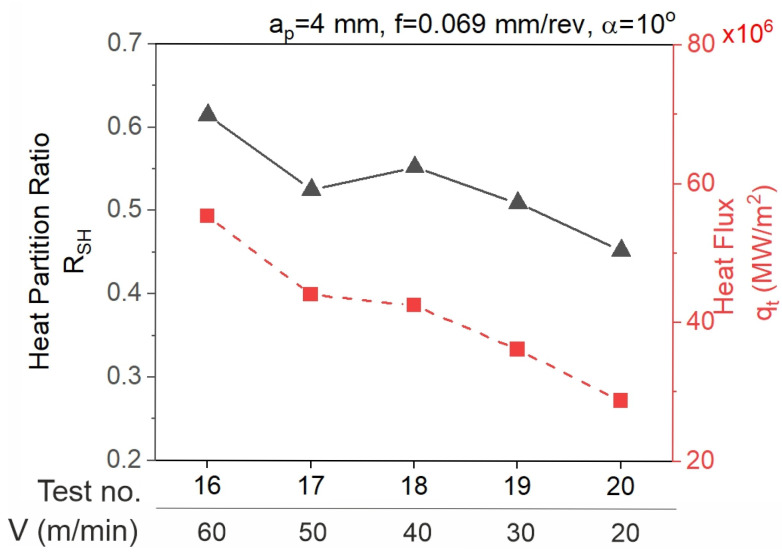
The impact of the cutting parameters on the heat partition ratio and the heat flux entering the tool.

Figure 16 presents the relationship between the heat partition ratio and the heat flux entering the tool and the cutting parameters. The analytically determined values of the heat partition ratio as well as the heat flux propagating between the chip and the tool increase as the cutting speed increases.

**Figure 17:**
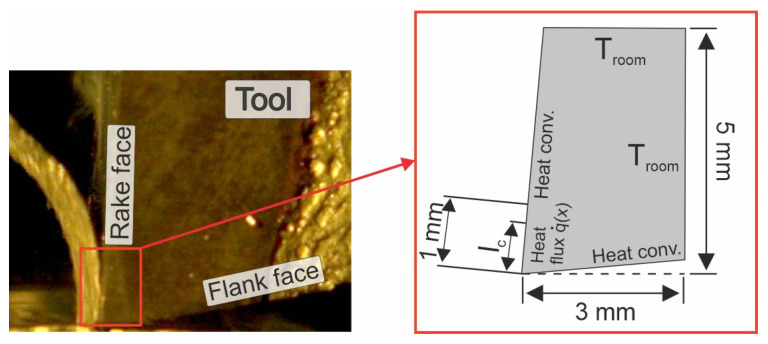
The boundary conditions used in MATLAB PDE.

The heat flux flow to the tool was modeled, and the temperature in the tool was determined using the MATLAB PDE Toolbox calculation package. The boundary conditions used in MATLAB PDE and the geometry are presented in Figure 17.

The following assumptions were used in the proposed method:

○ Tool geometrical dimensions were according to Figure 17;
○ Heat flux propagation was considered only on the rake face, within the chip–tool contact area, according to Figure 17;
○ Dor the remaining part of the rake face and for the flank face, the convection coefficient used was h_conv_ = 10 W/m^2^;
○ The temperature set for the outer tool edges was T_room_ = 20 ^°^C.

The modeling of the distribution of the temperature fields in the tool tip and in the chip used differential equations supplemented by the uniqueness conditions of the solution. For the case of orthogonal turning, the assumed model was a flat two-dimensional model for which, at point P(x,y), temperature T(x,y,t) is determined by a continuous function depending on coordinates x, y and time t.

The problems as well as heat transfer and conduction are described with a partial differential equation:

(17) $$\rho c_{p}\frac{\partial T}{\partial t}=\nabla \cdot (k\nabla T)$$

where T—temperature on the determined section of the tool rake face, *ρ*—density, *c_p_*—specific heat, *k*—thermal conductivity.

**Figure 18:**
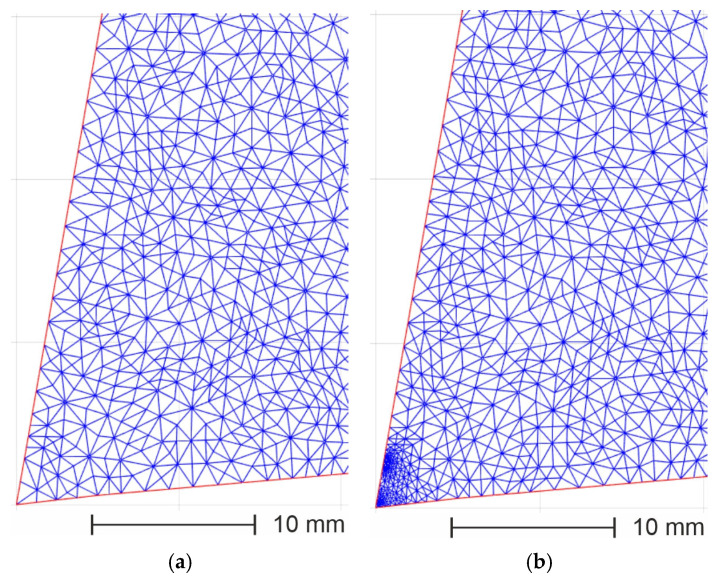
The cutting tool geometries with the superimposed FEM mesh before (**a**) and after (**b**) adaptation.

Figure 18 presents the cutting tool geometries with the superimposed FEM mesh before and after adaptation. The mesh densification in the chip–rake face contact place was implemented by modification of the mesh H_grad_ parameters.

The following assumptions were made in order to estimate the errors of the built simulation model:

- The boundary conditions were constant, according to Figure 17;
- The superimposition and densification methods of the FEM mesh on the tool significantly affected the calculation time.

An excessively dense mesh for the entire tool area extended the calculation time to 120 s for each test. The optimum mesh adaptation variant is presented in Figure 18. Such an approach allowed the calculation time to be reduced to 30 s.

The input parameters of the thermal conductivity model for the tool, the workpiece, and the thermal diffusivity for the workpiece were determined using the equations in (Table 8) for a temperature of 550 °C. In order to estimate the errors of the simulation model, the behavior was analyzed for the input parameters in the 450–650 °C temperature range. The resultant temperature values calculated by the model were in the ±15% range versus the results obtained for the temperature of 550 °C.

**Figure 19:**
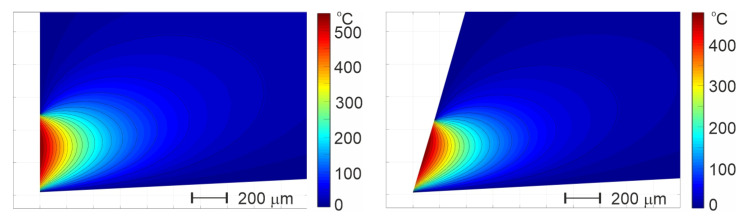
Examples of the temperature distribution on the cutting tool surface, obtained with MATLAB PDE Toolbox.

Examples of the thermograms presenting the temperature distribution on the cutting tool surface, obtained with the MATLAB PDE Toolbox for test No. 1 (α = 0°) and test No. 7 (α = 10°) are shown in Figure 19 below.

**Figure 20:**
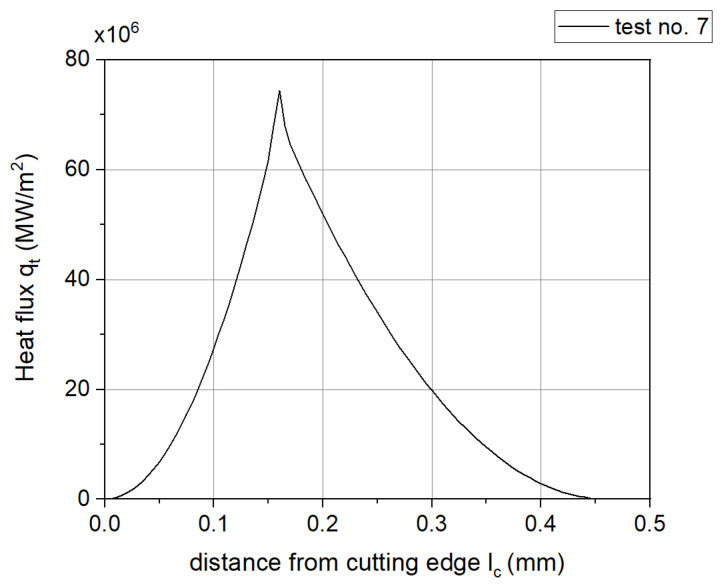
The distribution of the heat flux entering the tool.

An example of the distribution of the heat flux entering the tool for test No. 7 is presented in Figure 20.

**Figure 21:**
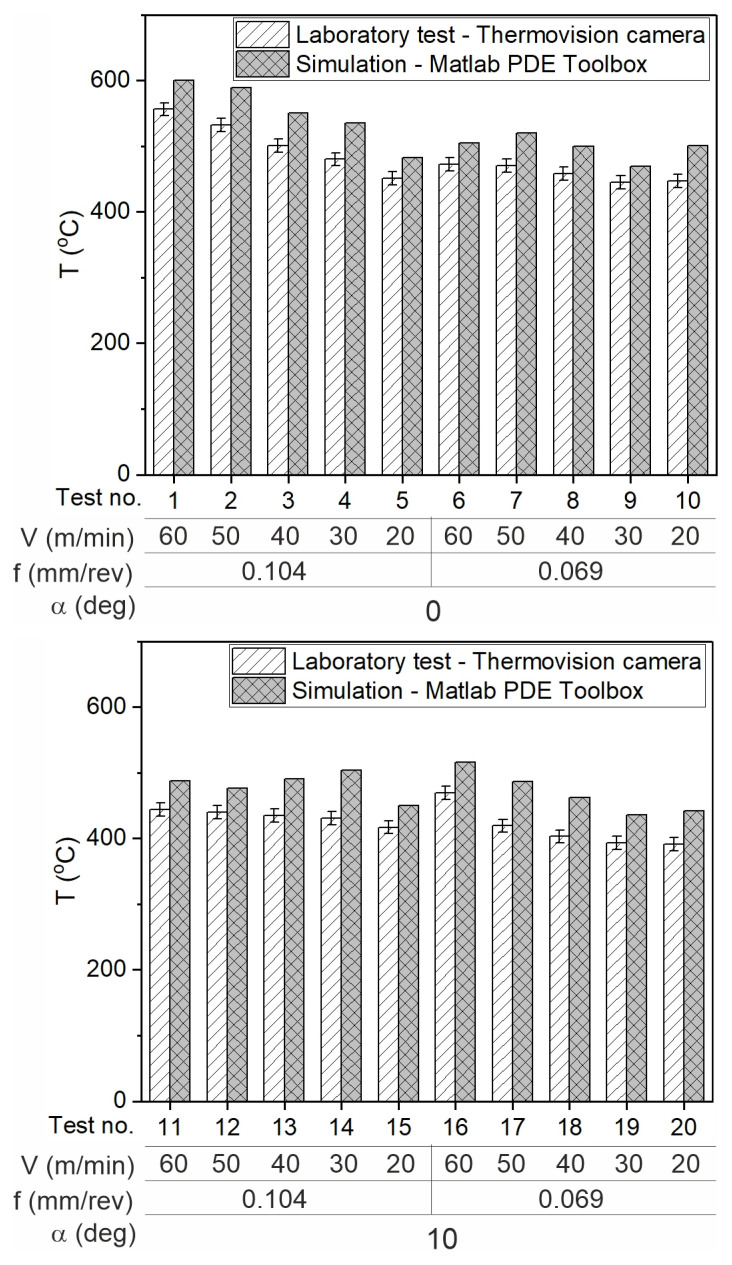
The comparison of the experimental and simulation results of the average contact temperature.

Figure 21 presents the temperature values in the contact zone, measured experimentally and calculated. The experimental values are lower than those calculated, but the difference is small, ranging from 24 °C to 53 °C for an angle of 0 degrees, and from 33 °C to 73° for an angle of 10 degrees, which is a 17% difference, indicating a good fit of results and confirming the correctness of the adopted model.

**Figure 22:**
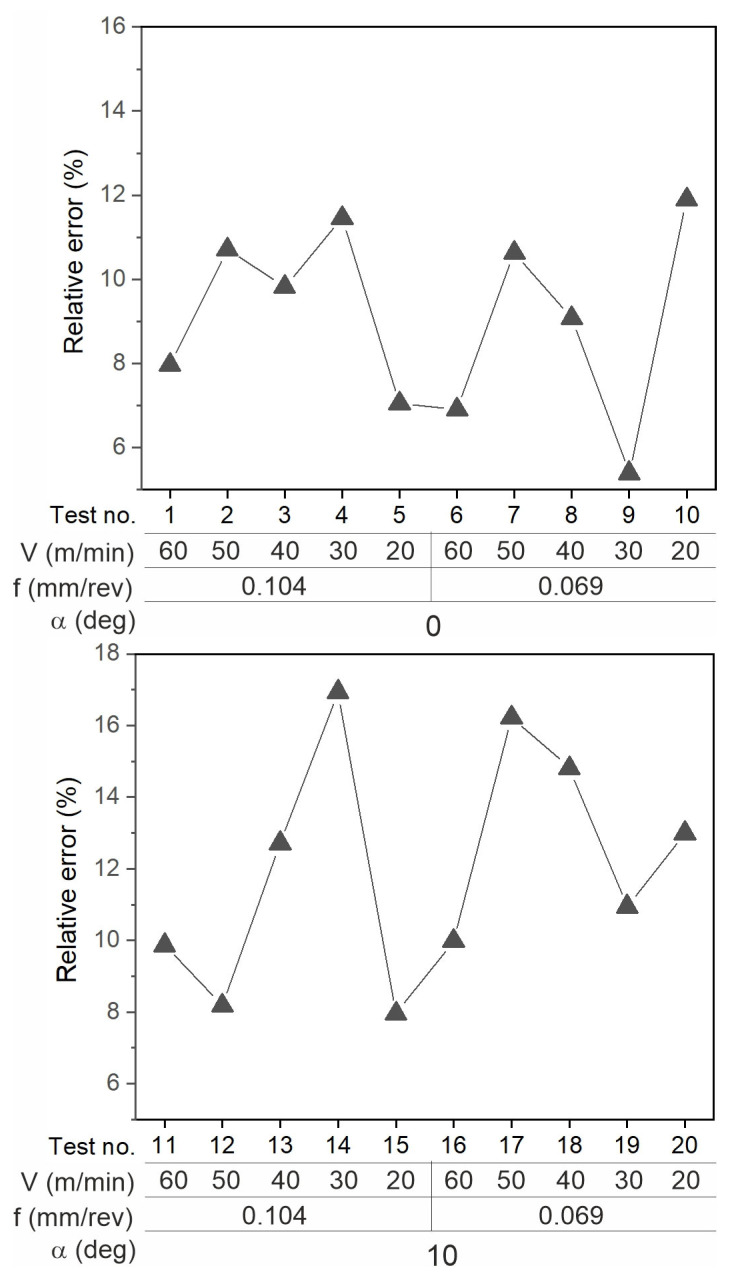
Relative error of measurement.

The differences between the experimental and simulation results (maximum temperature values) are presented as the relative error in Figure 22. The difference was determined using the formula D = abs(T_PDE_ − T_IR_)/T_IR_, where T_PDE_ is the maximum temperature determined in the simulation, and T_iR_ is the temperature determined experimentally.

## 5. Conclusions

Based on the results of the calculations and experimental work, the following conclusions were drawn:

○ The presented method is an original approach for the quick determination of the temperature on the tool rake face. The input data used in the method are the experimentally measured values of the cutting forces, the rake angle, and the cutting parameters.
○ The model has some limitations. It is an orthogonal model of a 2D cutting process. In addition, it is based on the average heat partition ratio according to Shaw’s method and does not account for the tool’s core temperature.
○ With a correctly calibrated model, differences may result from the fact that the thermovision camera measures the flank of the tool where the temperature is lower than in the central part of the rake face.
○ The experimental values of the contact temperature are lower than those calculated, but the difference is small, ranging from 24 °C to 53 °C for α = 0°, and from 33 °C to 73° for α = 10°, which is a 17% difference, indicating a good fit of the results and confirming the correctness of the adopted model.
○ The heat partition ratio for the tool (1-R_SH_) decreases as the cutting speed increases. The greatest value of the ratio was determined in test 5 (0.596) and the smallest value in test 6 (0.383).
○ The minimum heat flux value propagating to the tool was determined in test 20 and was equal to 28.680 MW/m^2^, which was 38.5% of the maximum value (in test 1, 74.452 MW/m^2^).
○ In addition, the model can be further improved by including reliable models of heat division and the tool core temperature in the algorithm.

## Figures and Tables

**Table 1:** The chemical composition of Ti-6Al-4V alloy.

Symbol	Ti	C	Fe	N	Al	O	V	H	Other
%wt.	bal	0.08	0.03	0.05	5.5–6.75	0.20	3.5–4.5	0.015	0.40

**Table 2:** Range of cutting parameters.

Symbol	Cutting Parameters	Parameter Values					
A	*f* (mm/rev)	0.069			0.104		
B	*V* (m/min)	20	30	40		50	60

**Table 3:** Plan of cutting tests.

Test No.	*F* (mm/rev)	*V* (m/min)	*a_p_* (mm)	*α* (degs)
1	0.104	60	4	0
2	0.104	50	4	0
3	0.104	40	4	0
4	0.104	30	4	0
5	0.104	20	4	0
6	0.069	60	4	0
7	0.069	50	4	0
8	0.069	40	4	0
9	0.069	30	4	0
10	0.069	20	4	0
11	0.104	60	4	10
12	0.104	50	4	10
13	0.104	40	4	10
14	0.104	30	4	10
15	0.104	20	4	10
16	0.069	60	4	10
17	0.069	50	4	10
18	0.069	40	4	10
19	0.069	30	4	10
20	0.069	20	4	10

**Table 4:** The values determined during the cutting tests.

Test No.	*F_c_* (N)	*F_t_* (N)	*ϕ* (degs)	*τ*_1_ (MPa)
1	924.3	687.2	26.68	558.4
2	938.0	699.0	26.65	565.9
3	963.5	724.5	26.53	578.1
4	976.3	746.9	26.29	579.7
5	984.0	760.2	26.16	580.8
6	705.0	642.3	23.83	564.2
7	730.2	677.3	23.58	577.3
8	740.0	685.5	23.59	585.5
9	762.2	685.0	24.03	615.5
10	781.0	687.1	24.33	639.7
11	762.1	430.2	30.28	534.8
12	774.3	440.3	30.19	541.4
13	782.5	449.1	30.07	544.6
14	796.3	468.2	29.77	547.5
15	810.0	487.2	29.49	550.5
16	584.8	451.8	26.16	520.3
17	589.3	460.4	26.00	520.7
18	598.8	475.0	25.79	524.1
19	596.3	480.2	25.58	517.1
20	601.3	489.3	25.43	518.0

**Table 5:** The experimentally determined average values of the chip flow speed and temperature.

Test No.	$V_{c}$ (m/min)	$T_{AVG}$ (°C)
1	30.2	556
2	25.1	532
3	20.0	501
4	14.8	480
5	9.8	451
6	26.5	472
7	21.8	470
8	17.5	458
9	13.4	445
10	9.0	447
11	32.3	444
12	26.8	440
13	21.3	435
14	15.8	431
15	10.4	417
16	27.5	469
17	22.8	419
18	18.1	403
19	13.4	393
20	8.9	391

**Table 6:** The values determined based on the proposed algorithm.

Test No.	$P_{0}$ (MPa)	$\mu_{a}$	μ
1	1344.3	0.743	0.908
2	1361.2	0.745	0.913
3	1386.2	0.752	0.931
4	1381.3	0.765	0.969
5	1378.9	0.773	0.992
6	1251.1	0.912	1.504
7	1269.7	0.910	1.496
8	1288.7	0.926	1.574
9	1373.5	0.928	1.360
10	1440.9	0.880	1.370
11	1098.7	0.823	0.908
12	1108.5	0.828	0.918
13	1110.2	0.835	0.931
14	1103.3	0.853	0.967
15	1096.9	0.870	1.001
16	893.8	0.879	1.203
17	887.6	0.896	1.230
18	883.9	0.902	1.257
19	862.7	0.916	1.280
20	857.6	0.925	1.310

**Table 7:** The analytically determined length of the chip contact with the tool face.

Test No.	$l_{c}$ (mm)	$l_{p}$ (mm)
1	0.516	0.204
2	0.517	0.206
3	0.521	0.209
4	0.530	0.221
5	0.535	0.225
6	0.423	0.216
7	0.431	0.260
8	0.431	0.224
9	0.416	0.254
10	0.407	0.200
11	0.461	0.159
12	0.464	0.242
13	0.468	0.164
14	0.478	0.249
15	0.488	0.179
16	0.417	0.185
17	0.423	0.232
18	0.430	0.194
19	0.438	0.241
20	0.444	0.180

**Table 8:** Thermo- mechanical properties of tool and workpiece materials [32,33].

Material	Property	Equation or Value
WC-Co	Thermal conductivity $\left(Wm^{-1}K^{-1}\right)$	$\lambda_{T}=28.969+0.03386\cdot T+7.977\cdot 10^{-6}\cdot T^{2}$
	Specific heat (Jkg^−1^K^−1^)	$c_{p}=398$
	Density (kgm^−3^)	*ρ* = $11.2\cdot 10^{3}$
Ti-6Al-4V	Thermal conductivity $\left(Wm^{-1}K^{-1}\right)$	$\lambda_{W}=14.99+34.65exp\left(-6.57\cdot 10^{-3}T\right)$
	Thermal diffusivity ${(m}^{2}s^{-1})$	$\alpha_{W}=5.7\cdot 10^{-6}+1.53\cdot 10^{-5}exp\left(-5.3\cdot 10^{-3}T\right)$

**Table 9:** Calculated flux and partition.

Test No.	*R_SH_*	Heat Flux (MW/m^2^)	Heat Flux *q_c_* (MW/m^2^)	Heat Flux *q_t_* (MW/m^2^)
1	0.558	168.390	93.938	74.452
2	0.520	139.080	74.529	64.551
3	0.504	115.445	58.161	57.284
4	0.463	87.710	41.323	46.386
5	0.404	56.535	22.865	33.670
6	0.617	149.522	92.193	57.329
7	0.596	127.725	75.796	51.929
8	0.560	102.402	57.307	45.095
9	0.524	79.330	42.662	36.668
10	0.499	57.860	28.880	28.980
11	0.597	172.469	103.039	69.430
12	0.564	142.904	82.166	60.738
13	0.542	116.225	62.952	53.273
14	0.496	88.073	44.657	43.416
15	0.437	57.489	25.128	32.361
16	0.615	143.265	88.071	55.195
17	0.525	117.835	63.198	43.925
18	0.553	94.785	52.369	42.416
19	0.510	74.635	38.589	36.045
20	0.453	52.423	23.744	28.680

## Data Availability

The original contributions presented in this study are included in this article. Further inquiries can be directed to the corresponding author.
